# Social Media in the Urology Practice | Opinion: NO

**DOI:** 10.1590/S1677-5538.IBJU.2019.05.04

**Published:** 2019-01-29

**Authors:** Rodrigo Donalisio Da Silva, Jeffrey J. Leow, Zainal Adwin Abidin, Edgar Linden-Castro, Edgar Iván Bravo Castro, Leonardo Tortolero Blanco, Jeremy Yuen-Chun Teoh, Pablo Nicolas Contreras, Marcelo Langer Wroclawski

**Affiliations:** 1 Division of Urology Department of Surgery University of Colorado School of Medicine Denver CO USA Division of Urology, Department of Surgery, University of Colorado School of Medicine, Denver, CO, USA;; 2 Department of Surgery Division of Urology, Denver Health Medical Center Denver CO USA Department of Surgery, Division of Urology, Denver Health Medical Center, Denver, CO, USA;; 3 Tan Tock Seng Hospital LKC School of Medicine Nanyang Technological University Singapore Department of Urology, Tan Tock Seng Hospital, LKC School of Medicine, Nanyang Technological University, Singapore;; 4 Center for Surgery and Public Health Brigham and Women’s Hospital Harvard Medical School, Boston MA USA Division of Urologic Surgery and Center for Surgery and Public Health, Brigham and Women’s Hospital, Harvard Medical School, Boston, MA, USA;; 5 Department of Surgery Universiti Teknologi MARA Malaysia Department of Surgery, Universiti Teknologi MARA, Malaysia;; 6 Centro Médico Puerta de Hierro Zapopan Jalisco Mexico Centro Médico Puerta de Hierro, Zapopan Jalisco, Mexico;; 7 Servicio de Urologia Hospital Central Militar Mexico Servicio de Urologia, Hospital Central Militar, Mexico;; 8 Servicio de Urologia Hospital Imed Levante Alicante Spain Servicio de Urologia, Hospital Imed Levante, Alicante, Spain;; 9 S. H. Ho Urology Centre, Department of Surgery The Chinese University of Hong Kong Hong Kong S. H. Ho Urology Centre, Department of Surgery, The Chinese University of Hong Kong, Hong Kong;; 10 Hospital Alemá Buenos Aires Argentina Servicio de Urologia del Hospital Alemán. Buenos Aires, Argentina;; 11 Hospital Israelita Albert Einstein São Paulo SP Brasil Hospital Israelita Albert Einstein. São Paulo, SP, Brasil;; 12 Hospital Beneficência Brasileira de São Paulo São Paulo SP Brasil Hospital Beneficência Brasileira de São Paulo, São Paulo, SP, Brasil

**Keywords:** Social Media, Urology, Review [Publication Type]

## INTRODUCTION

Recently, Social Media (SoMe) has been one of the most important resources in communication. Unsurprisingly, the medical and scientific community started to utilize the available online platforms in order to facilitate communication, promote scientific knowledge and initiate partnerships with other Institutions.

A 2018 survey of more than 5000 physicians found that 71% of physicians under the age of 40, 50% of those aged 40 to 49 years old, and more than 30% of physicians older than 60 years old regularly use SoMe (1). The American Urological Association survey in 2013 showed a high use of SoMe among its members (74%), of which fellows and residents consisted 86% and attendings 66% (2).

Online platforms such as Twitter, Facebook, and Instagram are available at no cost and provide the opportunity to interact with others around the world using your smartphone. The dynamic use of SoMe became attractive for physicians and scientists, specially allowing a communication pathway that was not available before among the most prominent experts and their peers.

Medical societies, medical meetings and conferences rapidly started to use SoMe to promote their events and engage participants in the discussions using hashtags that facilitated finding the specific content in social media platforms, and also provided metrics for the online engagement of their participants (3, 4).

Another online phenomenon is the spontaneous international workgroups such as #UroSoMe, #SoMe4Surgery, and others. These workgroups rapidly engaged specialists to discuss their clinical practice, to get advice from world experts, promote journal clubs, and also for research collaboration. In the first online event on Twitter of the #UroSoMe group, a reach of more than 2 million users was achieved, in more than 200 different geographic locations (5). Patients are also benefiting from the online discussions, having useful online resources for their education regarding their health issues, surgeries, providers review and other factors that can contribute for their decision-making.

SoMe use also came with controversy about ethics, copyrights and unprofessional use per health care providers and patients, medical associations, and online communities. Recommendations on the appropriate use of SoMe have been published before (6), however, the legislation regarding social media use of scientific content still varies worldwide. The use of pictures, photos, documents, patient’s body images or even radiology studies without proper patient consent has been criticized.

In this manuscript we will review potential pitfalls and harms of social media use, focusing in the health care aspect.

## Defining characteristics of SoMe

The first challenge concerning SoMe is to define its characteristics and to know all the available platforms, with their own particularities, so that issues can be properly addressed and compared. SoMe can be classified into two large groups (7):

1. Horizontal Social Networks: Mostly common available SoMe, where any user can join and participate, without having a priori common characteristics. Examples: Facebook, Instagram or Twitter.
2. Vertical Social Networks: Users looking to have common interests. These social networks serve one or several specific purposes at a professional level: employment, networking, travel, etc. Examples: LinkedIn, TripAdvisor, Soundcloud, Spotify, Vimeo, etc...

The use of SoMe can vary based on the user purpose. This includes the general user that may be joining SoMe for entertainment only, but several other aspects must be considered knowing that this is also used by corporations, marketing, branding, and others. Below we listed the most common uses for SoMe (8):

- Entertainment: SoMe started with the purpose of entertainment. The users are usually looking for topics related to their personal interest, such as sports, movies, books, travel and others. Content that users find interesting tends to generate likes and shares.
- Information: SoMe can be a great source of information, if the right sources are used. There is no editor on the shared content and inaccurate information can be found. Misinformation may even promote misunderstandings, propagate disbelief in healthcare professionals and lead to non-evidence based movements such as the anti-vaccine campaign.
- Personal contacts: To find and connect with those you already know, like family and friends. SoMe facilitates the contact not only by those that are geographically distant but also for those that cannot see each other often.
- Professional contacts: Professional contacts in your area of work, online networking and meetings. Initial online collaborative efforts via SoMe may eventually bear fruit and lead to formal professional relations.
- Online community: Online communities are created with common interests and to discuss related topics. It can take time to develop an online community.
- Web traffic: Sharing articles, topics, pictures and web links is often used to direct users to other websites. Trusting the source is the key for followers to take the step to research further and click on the shared link.
- Advertising: SoMe became huge for online advertising of brands, services or products. SoMe allowed segmenting users facilitating the achievement of a targeted audience.
- Branding: A new brand in SoMe emerge daily. Multiple publications, linking to other people, responding to followers and continuous interaction with the audience becomes a brand.
- Recommendation channel: When users like a product or service of a brand, they are likely to recommend it online. When researching for a new product or service, it is very common nowadays for users to research online about product or company reputation before buying. Most online services will follow up purchases with the intent that users share their experience with products. Multiple websites emerged only with the purpose to review and recommend products.

## MOST POPULAR SOME PLATFORMS (8)

### Facebook

Currently, Facebook is the SoMe with most users in the world, being an easy-to-use platform, allows different posts such as videos, images, or texts. Last year Facebook was involved in lack of security of users data. A wide variety of users of different ages are connected to Facebook. Facebook is the favorite social network for Millennial and Generation X. For Generation Z, the percentage Facebook users diminish in favor of other networks such as Instagram.

### Whatsapp

WhatsApp is one of the biggest instant message applications. It is the favorite choice to communicate among Millennials (40%) due to the facility and agility in communication. More than 80% of users connect through a mobile device. Apart from private messages, you can also create groups with several users.

### Youtube

The Youtube video platform is the third most commonly used social network. YouTube has a great capacity of interaction with other networks. YouTube allowed the emergence of digital influencers. It is also one of the fastest growing in number of users and is one of the best rated along with Instagram and Spotify. Young users are among those who consume the most audiovisual content. 43% of users between 16 and 23 years old follow at least one digital influencer through YouTube. In Urology, the most common use is sharing of surgical videos highlighting techniques and operative procedures.

### Instagram

In fourth position and following closely is Instagram. Younger users consider it the most important and relevant social network. Like Youtube, it is among the younger generations (between 16 and 23 years old) and for the second time in a row it is one of the SoMe that most attracts new users. The platform has been able to integrate the options of photography and video in a simple and attractive way. Brands have already captured this trend and are selling their products or services integrated among the publications of their acquaintances.

### Twitter

Twitter is a social network of microblogging, i.e. a network to publish, share, exchange information, through brief comments in text format, with a maximum of 140 characters, called Tweets. These tweets are displayed on each user’s main page. Twitter is considered the most important real-time communication nowadays. Users can subscribe to the Tweets of others, and it has the attraction of quickly updating the status from portable devices, such as smartphones. Important news are shared worldwide in real time. It can also be accessed from a PC, a laptop or tablet.

### LinkedIn

LinkedIn, unlike the latest SoMe, does not seem to have gained much traction among younger users. The LinkedIn social network seeks professional profile helping to connect professionals of different geographic location. It is also used by headhunters for recruitment.

The medical and scientific community took advantage of SoMe plateforms to colaborate and share knowledge to their peers and to general population. However, some aspects of SoMe use by these comunnites needs to be clarified to protect patients and the online community.

## PITFALLS AND CAUTIONS

### Scientific aspect of SoMe use

It becomes very common among physicians to share conferences, lectures and slides content on SoMe without certifying if there are any copyrights that must be respected. Some of the most difficult resources to reclaim copyrights is pictures of slides shared during meetings and conferences. Despite being prohibited in most conferences, it is almost impossible to refrain attendants or conference delegates from taking pictures and videos from the audience. Nevertheless, in the urological community, the American Urological Association (AUA) and the European Urological Association (EUA) websites stated that taking pictures or screen captures, or reproducing any materials without informed consent from authors and the society is not allowed. Most scientific journals require authors to sign a copyright release when publishing a manuscript. By doing this, authors cannot share or reproduce the content of the manuscript without journal permission.

We are facing a dilemma regarding the contents that could be reproduced and shared through SoMe. All intellectual property and information shared in SoMe should be followed by proper citations to avoid issues.

National and continental medical associations should provide education to members regarding SoMe in order to avoid and possible legal consequences of inflicting copyrights.

Sharing manuscripts or parts of manuscripts online is also common practice among physicians. However it generally involves a copyright agreement between the corresponding author (on behalf of the rest of the authors) and the journal publisher. Each journal publisher has its own copyright regulations and sharing policies and a list of links has been collated to allow users to quickly access each publisher’s sharing policy at <https://www.howcanishareit.com>. Although it does not reveal any specific rules pertaining to SoMe, generally the full article cannot and should not be shared publicly.

Conversly, journal publishers are increasingly recognizing the role of SoMe to help their authors disseminate research findings quickly. This is evidenced by the fact that most urology journals have an official Twitter account through which recently published articles are shared. Some journals, such as European Urology, even have a requirement for authors to submit a 160-character Tweet along with the manuscript for consideration. Although the correlation between Twitter mentions and subsequent citations has not been demonstrated (9, 10), there is a belief that sharing on SoMe may improve an article’s overall visibility and encourage more to visit the link embedded in the tweet (11, 12).

We recommend urologists to share a link of their article (either PubMed/MEDLINE or Digital Object Identifier [DOI]), with a screenshot of the title/abstract as an image. We do not recommend sharing the entire publication or it’s .PDF files on social media publicly due to potential copyright rules.

### Physicians perspective of SoMe use

It must be taken that SoMe is an open environment where everything published will be public domain, which means that other physicians, patients and the general population will have access to that. A major risk associated with the use of SoMe is the posting of unprofessional content that can reflect unfavorable bias or self-promotion.

A survey carried out by the Pew Research Internet Project found that 72% of the patients who responded to it have searched for some information in the social media (13). A similar concern is the exploitation of the patient in online communities that are influenced by marketing. Greene et al. found that a significant proportion (27%) of posts on Facebook about diabetes management in support groups appeared to be promotional, typically in the form of testimonials. Many of these recommendations are very vague and, in most cases, associated with a specific product without any disclosures (14). Another study identified that 69% of Youtube medical related videos suffer from bias and 73% are low quality videos that can lead patients to take erroneous decisions regarding their treatment. Some physicians take advantage and publish on SoMe health related information in their personal accounts. However, limitations of these informations found online are lack of quality and reliability (15).

A survey of the executive directors of American state medical boards revealed that at least one online professional violation had been reported in 92% (44/48) of responding jurisdictions (16). Inappropriate communication with patients (69%) and misrepresentation of credentials (60%) were the most common violations. As physicians we have to cross a line between our personal and professional opinion. Professionals should not claim treatments that cannot be substantiated or verified. Also, professionals should not advertise their services or results beyond medically verifiable data. Online discussions or posts which could be associated with financial conflicts of interest must be transparent and any conflict of interest should be disclosed.

Researchers analyzed the content of blogs written by health professionals, 11% contained product endorsement of specific healthcare products; none provided conflict of interest disclosures (17). Physician’s tweets may also involve product promotions (18). Of note, Federal Trade Commission regulations released in 2009 require material connections between advertisers and endorsers to be disclosed. A blogger who receives cash or payment to review a product is considered an endorser. In addition, both advertisers and endorsers may be liable for false or unsubstantiated claims made in an endorsement in the US (19), but in other countries there is still a lack of regulation.

### Patient’s perspective of SoMe use

Patients are the epicenter of medicine. Everything should revolve around patients and their wellbeing. Patients hand their lives and health to us with an enormous amount of private information. At the same time, as physicians, it is our duty to educate and share our experience with our peers and this is when it gets complicated. There are multiple factors that should deter increased use of SoMe from the patient’s perspective and this includes protection of privacy, “fake news”, credibility and source credibility.

In the era of technological mobility, patient’s privacy is often compromised. The appeal of medical imagery, especially in rare cases, puts the affected patient in a vulnerable position. The patient could no longer be protected from being exposed to the online crowds. Sharing innapropriate content is more prevalent among junior staffs, as suggested by a recent survey (20). Although patient’s privacy is protected by law, regional variations exist thus allowing loopholes to be utilized to ‘justify’ invasion of privacy. Furthermore, legislation is a tedious process and with the exponential growth of SoMe, the laws governing it will never be at par with the impact of SoMe. Despite the existence of consent documentation and lengthy discussions between physician and patient, the aspect of consent will always be imperfect due to the imbalance between legislation and SoMe outreach.

SoMe has become an indispensible part of the health-seeking-behavior complex. Health information is mostly sought through online and socially networked platforms (21). This is due to its nature of anonymity, ease of access, and abundance of information. Accuracy and relevance of information is often misjudged by the patients and this presents as hazard to them. The feature of SoMe is the remarkable, global importance of social experiences into the online domain (22). In this scenario, the phenomenon of “fake news” has come about. Anyone can upload seemingly good advice online despite it not having strong supportive evidence. The gold standard of medical research is the randomized controlled trial and while evidence-based medicine de-emphasizes anecdotal reports, SoMe tends to emphasize them, relying on individual expericences for collective medical knowledge (23). In addition, the Echo Chamber effect as proposed by Christopher Paul et al. points out how SoMe users often follow like-minded individuals thus allowing polarized opinions to gather momentum (24). This propagation of inaccurate information represents a serious hazard to the patient and could have fatal consequences.

As with any information, its source is often in question. Health information does not escape this fact. Increased use of SoMe blurs this line of identification. Health information from foreing countries are often quoted online and often times it is quoted as being from a “renowned” physician. Most times the reader has no idea on who this “renowned” physician is and this creates confusion amongst patients. Although there are ways to verify the identity of the physician in question, the widespread availability of this information is not readily available. There are efforts to streamline information and source with the World Health Organization leading a request to the Internet Corporation for Assigned Names and Numbers to establish a suffix for validated information (25) and this could pave the way for the safety of healthcare information online, but it is still a work in progress.

## CONCLUSIONS

SoMe revolutionized communication and healthcare professionals are included in this trend. These tools facilitate interaction among physicians, patients, associations, and organizations. Even though SoMe legislation varies broadly worldwide, physicians and scientists should be mindful of their posts, sharing only data with good scientific support with proper citations. Most importantly, patients’ privacy must be respected.
